# Parvovirus Minute Virus of Mice Induces a DNA Damage Response That Facilitates Viral Replication

**DOI:** 10.1371/journal.ppat.1001141

**Published:** 2010-10-07

**Authors:** Richard O. Adeyemi, Sebastien Landry, Meredith E. Davis, Matthew D. Weitzman, David J. Pintel

**Affiliations:** 1 University of Missouri-Columbia, School of Medicine, Columbia, Missouri, United States of America; 2 Salk Institute, La Jolla, California, United States of America; King's College London School of Medicine, United Kingdom

## Abstract

Infection by DNA viruses can elicit DNA damage responses (DDRs) in host cells. In some cases the DDR presents a block to viral replication that must be overcome, and in other cases the infecting agent exploits the DDR to facilitate replication. We find that low multiplicity infection with the autonomous parvovirus minute virus of mice (MVM) results in the activation of a DDR, characterized by the phosphorylation of H2AX, Nbs1, RPA32, Chk2 and p53. These proteins are recruited to MVM replication centers, where they co-localize with the main viral replication protein, NS1. The response is seen in both human and murine cell lines following infection with either the MVMp or MVMi strains. Replication of the virus is required for DNA damage signaling. Damage response proteins, including the ATM kinase, accumulate in viral-induced replication centers. Using mutant cell lines and specific kinase inhibitors, we show that ATM is the main transducer of the signaling events in the normal murine host. ATM inhibitors restrict MVM replication and ameliorate virus-induced cell cycle arrest, suggesting that DNA damage signaling facilitates virus replication, perhaps in part by promoting cell cycle arrest. Thus it appears that MVM exploits the cellular DNA damage response machinery early in infection to enhance its replication in host cells.

## Author Summary

Infection by DNA viruses can result in the induction of cellular DNA damage responses. These potent responses can present a block to the replication of certain viruses, but can also be utilized by others to their advantage. We show here that autonomous parvoviruses, single-stranded linear DNA viruses that infect many animal species including humans, can induce a strong DNA damage response following low multiplicity of infection. The response is dependent upon active viral replication, and the ataxia telangiectasia mutated (ATM) kinase is the main transducer of the signaling events in the normal murine host. Inhibitors of ATM restrict MVM replication and overcome virus-induced cell cycle arrest. Thus, DNA damage signaling in response to parvovirus replication facilitates virus replication, perhaps in part by promoting cell cycle arrest. Our results suggest that MVM exploits aspects of the cellular DNA damage response machinery early in infection to enhance its replication in host cells.

## Introduction

It has become increasingly clear that viruses, especially DNA viruses, can provoke DNA damage responses (DDRs) in infected cells, either in response to virally encoded proteins or to the large amount of foreign DNA produced during viral replication. These cellular responses are varied, and have the potential to impede or facilitate virus replication (reviewed in [1], [2]). In the case of adenovirus (Ad), the DDR constitutes a barrier that must be overcome in order for viral replication to proceed [3], [4], [5], [6], [7], [8]. In contrast, the small DNA tumor viruses, polyomavirus and simian virus type 40 (SV40), activate a DDR that facilitates their replication [9], [10], [11]. Herpesviruses show complex interactions with the DDR pathway: while there is reduced replication of herpes simplex virus (HSV) in the absence of some DNA damage proteins [12], the viral immediate early protein ICP0 also inhibits accumulation of certain repair factors at sites of DNA damage [13].

Parvoviruses are small non-enveloped icosahedral viruses that are important pathogens in many animal species including humans [14], [15]. They are the only known viruses of vertebrates that contain genomes of single-stranded linear DNA [16]. Minute virus of mice (MVM) is an autonomously replicating parvovirus which is lytic in murine cells and transformed human cells. The viral genome is approximately 5 kb and possesses inverted terminal repeats at each end which form different hairpin structures and serve as origins of replication [17]. MVM encodes two non-structural proteins: the larger non-structural phosphoprotein NS1 is required for viral replication, while NS2 plays important roles in the normal murine host but is dispensable for replication in many permissive transformed human cell lines [18].

In contrast to the DNA tumor viruses, parvoviruses cannot induce entry of cells into S-phase, and must wait for cells to cycle into S-phase to begin replication [17]. Other than the non-structural NS1 protein, they do not encode their own replicative machinery, and depend on cellular replication factors. Following initiation of parvovirus replication, there is a reorganization of the nucleus, leading to formation of distinct nuclear foci termed “autonomous parvovirus-associated replication” (APAR) bodies [19], [20], [21]. NS1 co-localizes with replicating viral DNA in APAR bodies, which also accumulate host replication proteins such as PCNA, RPA and DNA polymerases α and δ [19], [20], [21].

Several studies have suggested links between MVM, cell cycle and apoptosis [22], [23], [24]. MVM has been shown to cause cell cycle arrest in S and G2 phases, and this has been suggested to be mediated at least in part by stabilization of p53 [22], [23], [24]. Following MVM infection, there is a reduction in cellular DNA replication [25], and the endonuclease activity of NS1 has been suggested to cause nicks in cellular chromatin [23]. The related parvovirus H1 has been shown to cause apoptosis [26], [27], and in a recent report to induce phosphorylation of the histone variant H2AX [28]. In addition to the effects of the non-structural proteins, single-stranded parvoviruses present cells with distinct forms of foreign DNA during the infectious cycle. The related helper-dependent parvovirus adeno-associated virus (AAV) can induce a DDR. UV-inactivated AAV genomes delivered in very high amounts induce a robust cellular response [29], [30], and recent studies have suggested that AAV replication, in the context of helper Ad co-infection or Ad helper proteins, resulted in a unique DNA damage response which was not seen by infection with virus alone [31], [32]. Detailed investigation and interpretation of AAV induction of cellular DDRs during replication is complicated, however, by the potent effects of its helper viruses (Ad and HSV).

The cellular MRN complex consists of the Mre11, Rad50 and Nbs1 proteins, and acts as a sensor of double strand breaks (reviewed in [33], [34], [35], [36]). This complex is redistributed to DNA damage sites and recruits the ataxia telangiectasia mutated (ATM) kinase. In addition to ATM, two other phosphatidylinositol 3-kinase-like kinases (PIKKs) act as transducer kinases that communicate DNA damage signals to downstream targets in response to various stimuli: ATM-related (ATR), and DNA-dependent protein kinase (DNA-PK) [37], [38]. ATR is mainly activated following replicative stress [39], [40] while DNA-PK is mainly involved in DNA repair *via* the non-homologous end joining pathway (NHEJ) [41]. Once at the site of damage, these kinases phosphorylate histone H2AX [42], [43] and activate additional cell cycle checkpoint kinases [44], [45]. This leads to cell cycle arrest or induction of apoptosis in cases of severe DNA damage [46], [47].

In this report we have investigated the interaction between MVM and the cellular DNA damage response pathway. We show that MVM induces a robust DDR in infected murine, hamster, and transformed human cells. The cellular response requires replicating viral DNA. DDR sensor and response proteins accumulate in MVM replication centers, but at late times there is a proteasome-dependent loss of Mre11. We show that ATM is the primary kinase required for DDR signaling in response to MVM infection of rodent cells. The response observed with this autonomous parvovirus is distinct from that described for AAV in the presence of helper virus [31], [32], and appears to be more similar to that observed with papovaviruses. Chemical inhibitors of ATM restrict MVM replication and ameliorate virus-induced cell cycle arrest. MVM therefore appears to exploit the cellular DNA damage response to enhance its replication in host cells.

## Results

### MVM infection induces a DNA damage response in both murine and human cells

**Figure 1:**
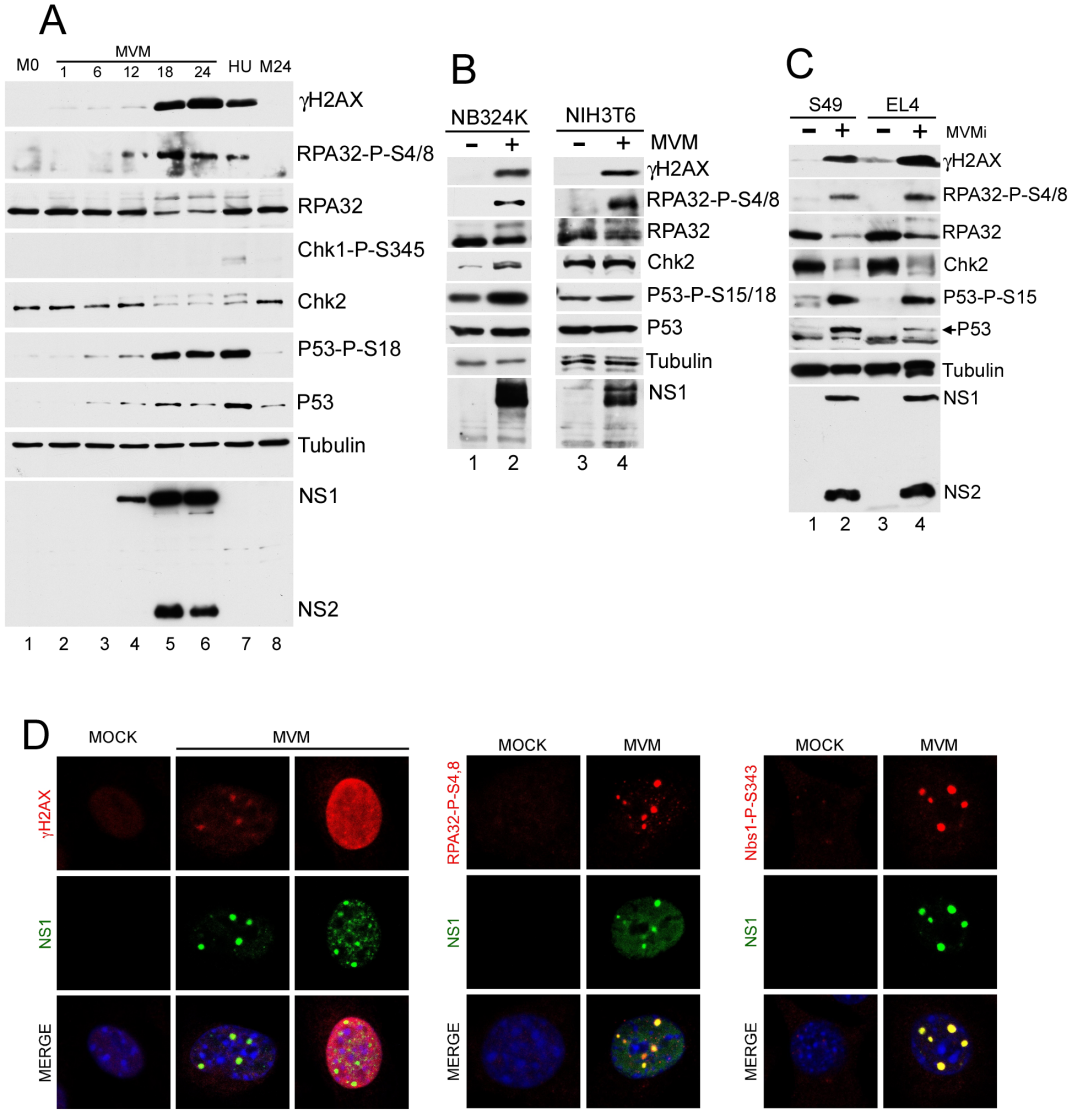
MVM infection induces a DNA damage response (DDR). (A) Time course of DDR following infection of A9 cells. A9 cells were para-synchronized in G0 as described in materials and methods. Cells were then mock-infected or infected with MVMp at an MOI of 7. As a positive control for the DDR, A9 cells were treated with 2 mM hydroxyurea (HU) for 12 hrs. Infected cells were harvested every 6 hrs over a 24 hr period and lysed in modified RIPA buffer. Protein content was measured using Bradford assay and equal amounts of protein were loaded in each well for immunoblotting. Western blot analysis was carried out using antibodies against NS1/2, tubulin, γH2AX, RPA32, Chk2, phosphor-Chk1, phosphor-p53 and p53. M0 and M24 represents mock samples at 0 hr and 24 hr time points respectively. (B) DDR following MVM infection in murine NIH3T6 cells and permissive human NB324K cells. Western blot analysis of virus and DDR proteins 24 h.p.i. of NIH3T6 cells and NB324K cells with MVMp. For detection of NS1, an antibody specific to NS1 alone was used. (C) DDR following infection with lymphocytic strain of MVM, MVMi. Western blot analysis of DDR proteins 24 h.p.i. of mouse S49 and EL4 lymphocyte cell lines with MVMi. (D) Cellular DDR proteins localized within APAR bodies. Para-synchronized A9 cells were infected with MVMp (MOI of 10) for 24 hrs before being fixed and processed for immunofluorescence. Cells were stained with the indicated antibodies to mark activated DDR proteins. Staining with an antibody to phosphorylated H2AX (γH2AX) was observed in distinct foci and also in a pan-nuclear pattern as described in the text. APAR bodies were detected with antibodies to NS1. Nuclei were stained with DAPI. All images were captured using an objective of 63×.

To examine whether MVM induced a DDR, para-synchronized murine A9 cells were infected at low multiplicity infection (MOI) with the MVM prototype strain MVMp. Phosphorylation of damage response proteins was detected by Western blotting and visualized by immunofluorescence (Fig. 1). Expression of the viral replicator protein NS1, a marker for viral replication, was detected by 12 hours post-infection (h.p.i.), and by 18 h.p.i. robust expression of both NS1 and NS2 could be observed (Fig. 1A, lanes 4–6). Co-incident with viral gene expression, we observed activation of a cellular DNA damage response that persisted throughout the time course monitored (Fig. 1A, lanes 4–6). We detected the appearance of phosphorylated H2AX (γH2AX), as well as p53 phosphorylated at S18 (S15 in human cells), and phosphorylated RPA32 (as identified with an antibody detecting phosphorylated S4/8 and by the concomitant decrease in electrophoretic mobility using antibodies to total RPA32). In addition, there was an increase in total p53 levels following MVM infection, consistent with a previous report [24]. The appearance of these DDR markers was similar to that seen following treatment with hydroxyurea (Fig. 1A, lane 7), a known inducer of the DDR [48], and absent from para-synchronized, mock-infected cells (Fig. 1A, lanes 1 and 8).

When examining downstream DNA damage effector kinases during MVMp infection, we observed phosphorylation of Chk2, a downstream target of ATM [49], [50], [51], as evident by an decrease in electrophoretic mobility (Fig. 1A, lanes 4–6), and confirmed by both reactivity with anti-Chk2-P-T68 antibody and phosphatase susceptibility (data not shown). In contrast, we did not detect phosphorylation of Chk1, a kinase typically phosphorylated at serine 345 in response to ATR activation [52], [53], [54], [55]. A significant DDR was also detected when re-infection was blocked by added neutralizing antibody in the media (data not shown), suggesting that a single round of infection was sufficient to induce the DDR. The DDR was also observed during infection of asynchronous A9 cells (data not shown).

We confirmed that activation of the DDR by MVM was not limited to the A9 cell line or MVMp strain. Additional permissive cell lines murine NIH3T6 and human NB324K were infected with MVMp and analyzed for DDR signaling. Infection of these cell lines resulted in the phosphorylation of H2AX, RPA32, p53 and Chk2 (Fig. 1B). Similar results were also seen following infection with the lymphotropic variant of MVM, MVMi, in the murine lymphocyte lines S49 and EL4 (Fig. 1C). Differences in the relative levels of NS1∶NS2 by MVMp and MVMi have been previously reported [56]. Taken together, these results demonstrated that autonomous parvoviruses induced a significant DDR in permissive murine and human cells.

### Components of the DDR localize to sites of MVM replication

MVM replication takes place in viral-induced APAR bodies, which contain RPA, DNA pol-α, pol-δ, and cyclin A, in addition to the viral replicator protein NS1 and replicating genomes [19], [20], [21]. The MVM-induced phosphorylated DDR proteins could also be detected by immunofluorescence at APAR bodies (Fig. 1D). After 24 h of MVMp infection in murine A9 cells, γH2AX staining was detected and primarily co-localized with NS1 at distinct APAR bodies that represent early stages of infection (Fig. 1D, left panel, second column). In cells with more diffuse NS1 patterns, that represent later stages of the infection cycle, we observed pan-nuclear staining of γH2AX (Fig. 1D, left panel, third column). The majority of infected cells also showed co-localization of NS1 with staining patterns for S4/8-phosphorylated RPA32, and the S343–phosphorylated form of the MRN complex component Nbs1 (Fig. 1D, center and right panels, respectively). Similar results were obtained in NB324K cells (data not shown).

**Figure 2:**
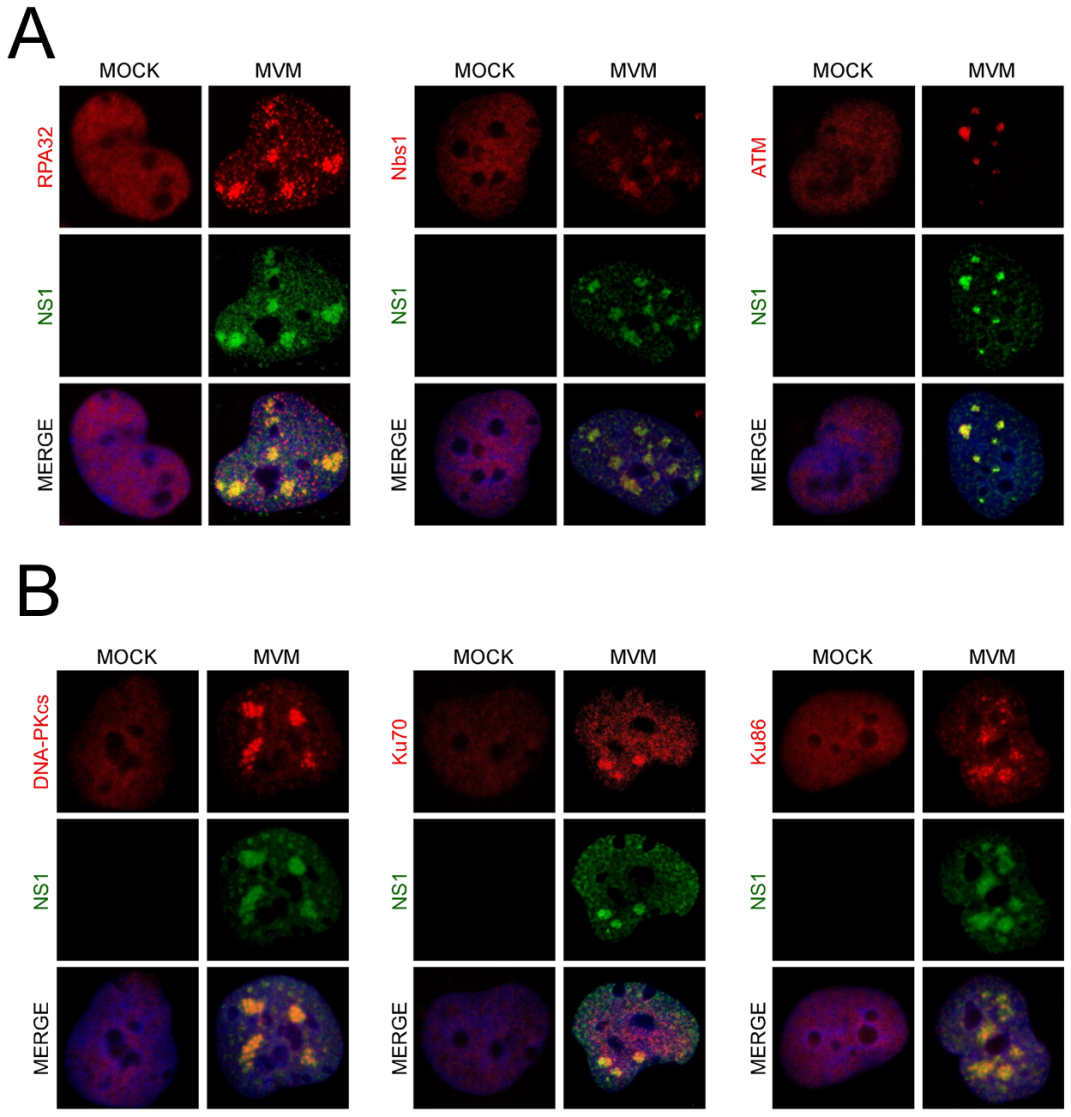
DNA repair proteins accumulate at MVM APAR bodies. (A) Repair proteins accumulate at APAR bodies. NB324K cells were infected with MVMp (MOI of 10) for 16 hr before being fixed and processed for immunofluorescence. Cells were stained with the indicated antibodies to mark DDR repair proteins. APAR bodies were detected with antibodies to NS1. Nuclei were stained with DAPI. All images were captured using an objective of 63×. (B) DNA-PK components localize to APAR bodies. NB324K cells were infected with MVMp (MOI of 10) for 24 hr before being fixed and processed for immunofluorescence. Cells were stained with the indicated antibodies. APAR bodies were detected with antibodies to NS1. Nuclei were stained using DAPI. All images were captured using an objective of 63×.

We also found that MVM dramatically redistributed bulk DDR-associated proteins within the nucleus of infected NB324K cells. In addition to the previously reported redistribution of RPA into APAR bodies [19] (Fig. 2A, left panel), we also found that MVM infection led to accumulation of Nbs1 into NS1-containing APAR bodies, in contrast to the diffuse nuclear staining pattern observed in mock cells (Fig. 2A, center panel). Nbs1 has been shown to recruit ATM to sites of DNA damage via its C-terminus [57], [58]. We examined ATM localization using the rabbit monoclonal antibody Y170, which has previously been validated for ATM immunostaining [59]. We detected endogenous ATM redistributed to APAR bodies in MVM-infected cells (Fig. 2A, right panel). In addition, we also observed redistribution of DNA-PK and its components Ku70 and Ku86 to MVM replication compartments (Fig. 2B). Taken together these results show that MVM replication leads to a reorganization and redistribution of DDR proteins to MVM sites of virus replication.

### MVM-induced DDR is associated with reduction in Mre11 levels

**Figure 3:**
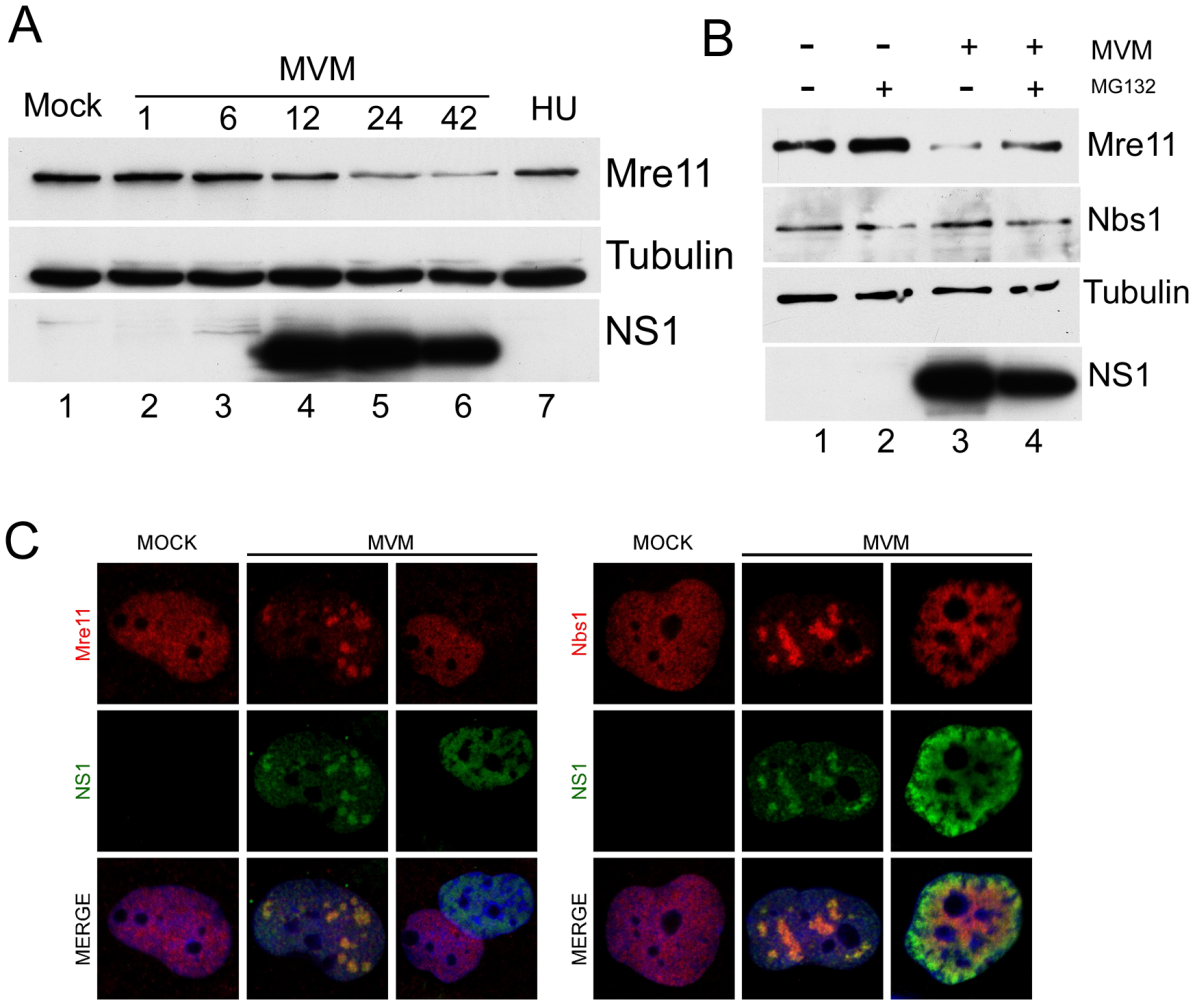
Mre11 but not Nbs1 is degraded in MVM-infected cells. (A) Mre11 levels were reduced late in infection. Western blot analysis of a time course of MVM infection of A9 cells shows reduction in the levels of Mre11 protein at 24 h.p.i but not in mock-infected (M) or hydroxyurea-treated (HU) cells. Tubulin served as a loading control. (B) Mre11 loss is proteasome-dependent. Mock-infected and MVM-infected A9 cells were treated with MG132 or DMSO at 24 h.p.i. for 6 hours. Western blots show reversal of Mre11 loss following treatment with MG132, whereas the levels of Nbs1 remain unchanged. (C) Mre11 loss examined by immunofluorescence. Confocal immunofluorescent microscopy comparing staining for Mre11 and Nbs1 in uninfected and MVM-infected NB324K cells. Both Mre11 and Nbs1 are found co-localized with NS1 in APAR bodies at early stages of infection but only Mre11 levels are specifically decreased at late stages of infection when NS1 is diffusely nuclear. All images were captured using an objective of 63×.

We observed that levels of the Mre11 component of the MRN complex, which is associated with ATM activation [33], [34], were reduced approximately 3-fold by 24 hrs following MVM infection of A9 cells (Fig. 3A). Loss of Mre11 was also reported following infection by SV40 [11], and adenovirus [8], [60]. This reduction, which was not seen during the DDR induced by hydroxyurea (Fig. 3A), was partially reversed in the presence of MG132, suggesting its loss was due to proteasome-mediated degradation (Fig. 3B). Surprisingly, and in contrast to the case for SV40 and adenovirus, no decrease was observed for other MRN components, such as Nbs1 (Fig. 1B). In addition, we did not observe any reduction in the levels of ATM or DNA-PK following MVM infection (data not shown). Immunofluorescence analysis demonstrated that Mre11 was redistributed to co-localize with NS1 in cells with distinct MVM APAR bodies, but staining was completely undetectable in cells that represent the late stages of MVM infection, with robust and diffusely nuclear NS1 expression (Fig. 3C, left panel). These assays also demonstrated the sustained presence of Nbs1 throughout infection (Fig. 3C, right panel). These results suggest the specific down-regulation of Mre11 at the late stages of MVM infection.

### MVM-induced DDR requires viral replication

**Figure 4:**
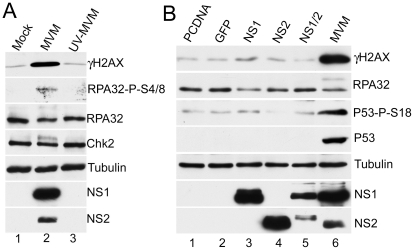
MVM-induced DDR requires viral replication. (A) Loss of DDR following UV-inactivation of MVM. MVM was UV-inactivated as described in materials and methods. A9 cells were mock-infected or infected with wild-type or UV-inactivated MVM at an MOI of 10. Western blot analysis of DDR proteins as well as NS1/2 and tubulin, of samples taken 24 hrs post-infection, revealed that UV-inactivated MVM did not activate the signaling pathways induced by wild-type MVM. (B) Expression of non-structural proteins is not sufficient for full DDR. Western blot analysis of DDR proteins 48 hrs following transfection of A9 cells with plasmids expressing MVM non-structural proteins as well as control plasmids pcDNA and GFP. MVM infection served as a positive control for the DDR, and tubulin was used as a loading control.

The MVM-induced DDR correlated with the onset of viral DNA replication (Fig. 1A). We thus attempted to determine which viral elements or functions induced the cellular response. As expected, infection with UV-inactivated MVM (calculated prior to treatment to infect at an MOI of 10) did not generate the viral non-structural replication proteins (Fig. 4A). When UV-MVM was compared to a parallel infection done at the same multiplicity with untreated virus, activation of the DDR proteins was not detectable (Fig. 4A, compare lane 3 to lane 2). These results suggested that neither the infecting virion itself, nor the incoming viral genomes, were sufficient to bring about a DDR at low MOI. This implicated either expression of the viral genes and/or viral replication as responsible for the induction of the cellular DDR.

To assess the contribution of MVM proteins to activation of the DDR, we transfected expression vectors for non-structural NS1 and NS2 proteins into A9 cells. Ectopic expression of NS1, NS2 or NS1+NS2 did not generate significant levels of DDR signaling (Fig. 4B), although expression of NS1 alone led to a slight increase in phosphorylated H2AX above background levels. The requirement for NS2 in activation of the DDR was further analyzed using the NS2-null host-range mutant MVM1989, which is restricted in murine hosts but grows to wild-type levels in human NB324K cells [18]. Infection with MVM1989 generated a DDR to levels similar to wild-type virus in the permissive cells, confirming NS2 was unnecessary to generate this response (data not shown).

These results suggest that viral replication is necessary for induction of the DDR, which cannot be accomplished efficiently merely by expression of MVM RNA or proteins. Since MVM replication is restricted to S-phase, it also follows that the DDR response that it induced is initiated during that phase of the cell cycle.

### ATM is activated during MVM infection and predominately mediates the DDR

**Figure 5:**
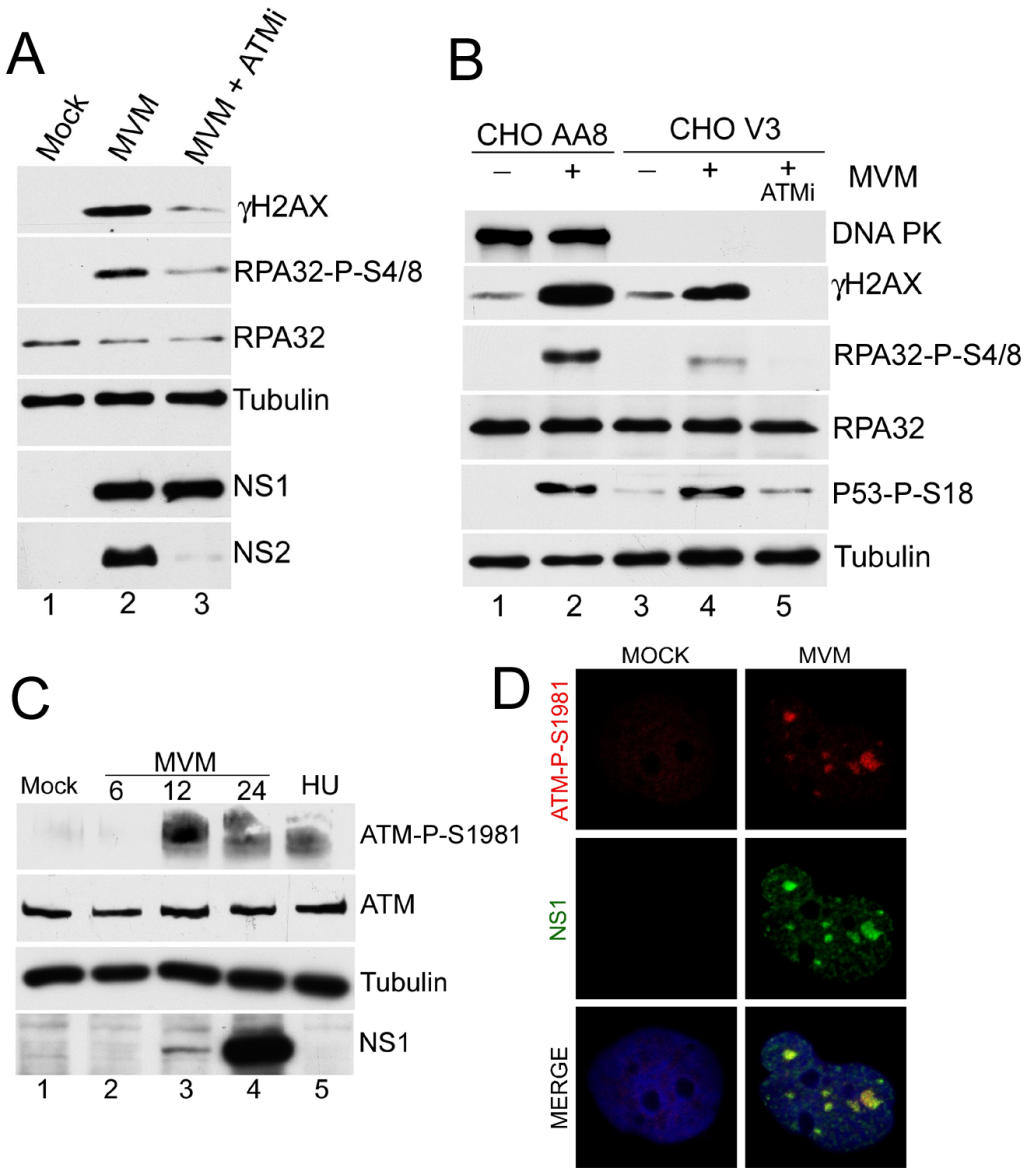
Activated ATM is predominantly responsible for signaling events during MVM infection. (A) ATM inhibitor reduces signaling events. Western blot analysis comparing DDR proteins at 18 h.p.i. of parasynchronized A9 cells infected with MVMp in the absence or presence of 7.5 µM ATM inhibitor KU 55933 (ATMi). (B) ATM-mediated signaling events seen in the absence of DNA-PKcs. Western blot analysis of DDR proteins following mock or MVMp infection of CHO-AA8 and CHO-V3 cells deficient in DNA-PKcs. Treatment with 10 µM ATM inhibitor KU 55933 (ATMi) blocked signaling in the CHO-V3 cells. (C) Activation of ATM following MVM infection. Western blot analysis shows ATM phosphorylation on S1981 following MVM infection of NB324K cells and hydroxyurea (HU) treatment. (D) ATM-dependent staining at APAR bodies. NB324K cells were infected with MVMp (MOI of 10) for 16 hr before being fixed and processed for immunofluorescence. Cells were stained with antibodies to NS1 and an antibody generated to phosphorylated ATM. Nuclei were stained with DAPI. All images were captured using an objective of 63×.

To address which cellular kinase(s) might be responsible for signaling activated during MVM infection, we analyzed the DDR in the presence of chemical inhibitors and utilized mutant cell lines (Fig. 5). Treatment of MVM-infected A9 cells with caffeine, which inhibits both ATM and ATR [61], dramatically reduced the DDR, while the DNA-PK inhibitor NU7026 [62], [63] reduced the DDR only minimally (data not shown). More specifically, the ATM inhibitor KU55933 [64] could inhibit DDR signaling as monitored by γH2AX and phosphorylated RPA32 (Fig. 5A, compare lane 2 to 3). The ATM inhibitor KU55933 and caffeine produced similar levels of inhibition of DDR signaling when compared in parallel (data not shown), suggesting that ATM was the primary mediator of this effect, and that ATR likely played only a minor or auxiliary role. Whether the reduction in the observed levels of the highly labile NS2 was a direct effect of ATMi, or merely reflective of less transcription template due to the inhibition of virus replication, is not known. When infected with MVM (as confirmed by expression of NS1 and NS2, data not shown) a CHO cell line deficient in DNA-PK (CHO V3) [65] responded with an efficient DDR, as evidenced by phosphorylation of H2AX, RPA32 and p53 (Fig. 5B), although the magnitude of the response was somewhat lower than the parent cell line CHO AA8 (Fig. 5B, compare lanes 2 and 4). Signaling in the DNA-PK-deficient cells was blocked by the ATM inhibitor, confirming that the DDR is ATM-dependent. Consistent with a role for ATM in the mediation of the DDR, phosphorylated ATM was detected in MVM-infected NB324K cells by Western blot analysis using an antibody generated to the S1981 residue auto-phosphorylated in ATM (Fig. 5C). Similar results were seen for A9 cells (data not shown). We used immunofluorescence to assess MVM-infected human NB324K cells for reactivity to this antibody against phosphorylated ATM. Staining with this antibody co-localized with MVM NS1 in viral replication APAR bodies (Fig. 5D). Taken together, these results demonstrated that ATM is the primary mediator of the DDR to MVM infection.

### ATM kinase activity is required for efficient MVM replication

**Figure 6:**
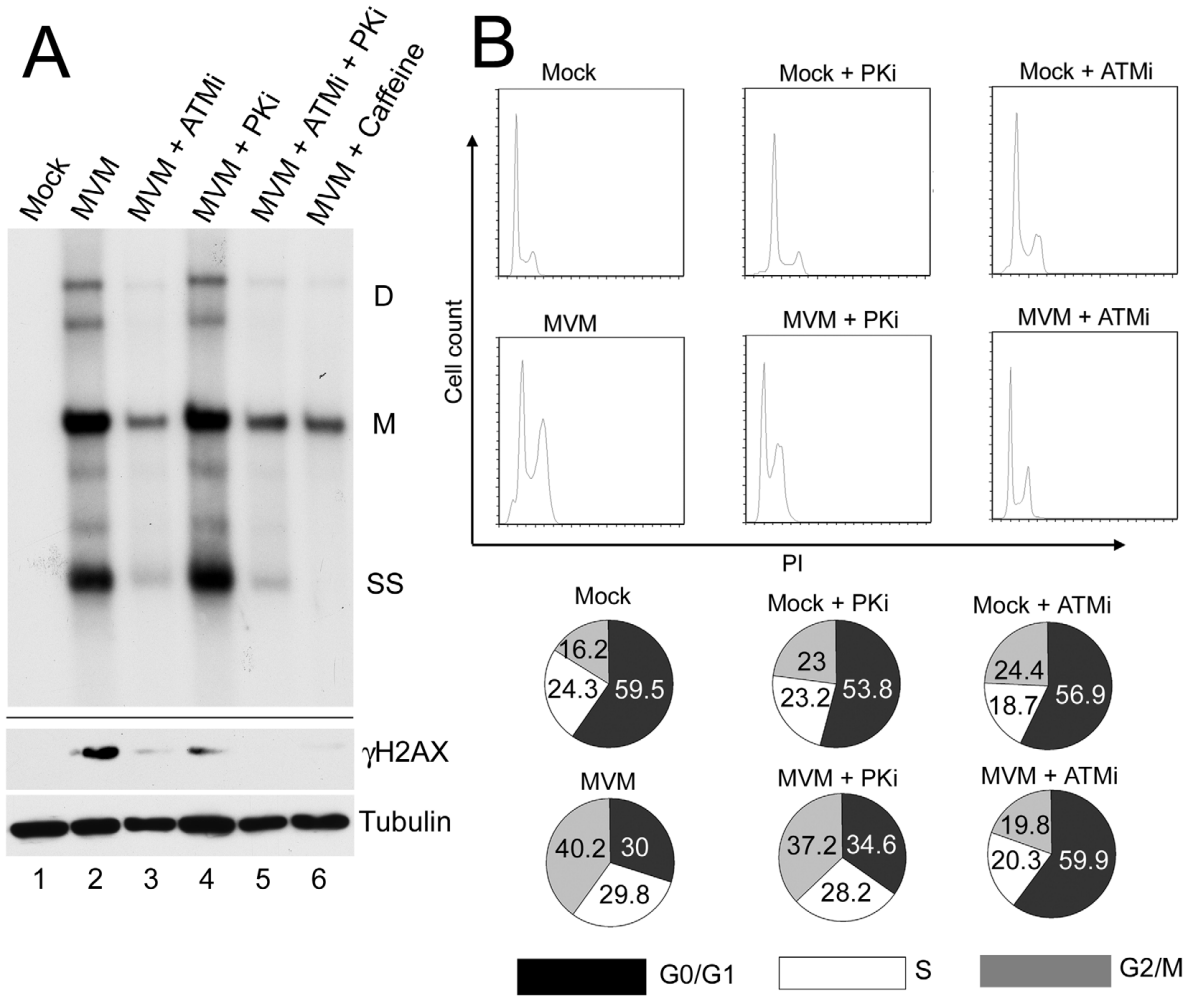
ATM kinase activity is required for efficient MVM replication. (A) Reduction of virus replication following inhibition of ATM kinase activity. A9 cells were pre-treated with DMSO or 7.5 µM ATM inhibitor (ATMi), 10 µM DNA-PK inhibitor (PKi), ATM and DNA-PK inhibitors in combination (ATMi+PKi), or 2.5 mM caffeine for 30 min prior to MVM infection at an MOI of 10. Lysates were split in half and used for Southern and western blot analysis. Southern blots were carried out as described in materials and methods and shown in the upper panel. DNA content was measured and equal amount of DNA loaded in each well. The blot was hybridized with a radiolabeled MVM probe and replicative intermediates of single-stranded DNA, SS; monomer, M; and dimer, D; are indicated to the right. Western blot analysis was also carried out as described and shown in the lower panel. The reduction in γH2AX signal confirmed inhibition of signaling by the inhibitors. (B) Reversal of cell cycle arrest following ATM inhibition. FACS analysis was carried out as described in materials and methods. Cells were pre-treated for one hour with 7.5 µM KU55933 (ATMi), 10 µM NU7026 (DNA-PKi) or DMSO vehicle before being mock-infected or infected with MVMp at an MOI of 10 for 28 hours. Representative histograms showing cell counts plotted against propidium iodide (PI) intensity are shown. The percentage of cells in each cycle stage was quantified using Flowjo software and shown in pie charts. Numbers are averages from two independent experiments.

We tested the importance of the DDR for MVM replication by assessing accumulation of viral DNA in the presence and absence of kinase inhibitors using Southern blotting of DNA extracted from infected cells (Fig. 6A). Pre-treatment of murine A9 cells with the ATM inhibitor KU55933 significantly reduced MVM replication, compared to untreated cells and cells treated with the DNA-PK inhibitor NU7026 (Fig. 6A, compare lane 3 to lanes 2 and 4). The reduction achieved by the ATM inhibitor was similar to that obtained by pre-treatment with caffeine, or the combination of NU7026 and KU55933 (Fig. 6A, lanes 5 and 6). These results demonstrate that the ATM kinase activity, which mediates the DDR response to MVM infection, facilitates efficient virus replication.

We assessed the effect of the DDR on the cell cycle arrest induced by MVM infection (Fig. 6). Parvovirus replication is dependent on cells cycling through S-phase, and is known to induce a cell cycle block past this point, either in late S-phase or in G2/M [22], [24]. Cells were pre-treated with the inhibitors to ATM and DNA-PK and then infected with MVM. As expected, treatment with the ATM inhibitor alone did not induce a cell cycle block that might inhibit MVM infection (Fig. 6B). Treatment of infected cells with the ATM inhibitor, however, did overcome the replication-dependent MVM-induced block at G2/M (Fig. 6B). In contrast, the DNA-PK inhibitor NU7026 had a negligible effect. These results suggested that the MVM-induced DNA damage response facilitates viral replication, perhaps in part by promoting cell cycle arrest.

## Discussion

In this study we investigated interaction of the autonomous parvovirus MVM with the cellular pathways that respond to DNA damage. We have shown *via* Western blots and immunofluorescence that MVM replication induced a strong DNA damage response, characterized by phosphorylation of H2AX, RPA32, p53, Nbs1, Chk2 and ATM. The majority of these phosphoproteins accumulated at MVM-induced APAR bodies in the nuclei of infected cells. The DDR was observed following MVM infections of permissive cells that were transformed and untransformed, and in cells of murine, hamster and human origin. In addition, the lymphotrophic strain of MVM also led to robust signaling events following infection of murine lymphocyte lines. Together these results demonstrate that infection with the autonomous parvovirus MVM leads to activation of the cellular signaling pathways that form the DDR. Our study is the first to report induction of a DDR by an autonomous parvovirus. Similar results have recently been observed for the autonomous parvovirus minute virus of canine (MVC) (J. Qiu, personal communication).

Comparing cellular responses to different viruses is often revealing about virus-host interactions in general, and can provide insights into fundamental cellular processes. Replication of the related helper-dependent parvovirus AAV, which also consists of a single-stranded linear DNA genome, can occur in the absence of helper following UV-treatment of host cells [66]. AAV has recently been shown to induce a unique DDR [31], [32], and, in contrast to what we report here for MVM infection, the DDR signaling events activated in response to AAV replication appear to be predominantly mediated by the DNA-PK kinase [31], [32]. Interpretation of the DDR induced during AAV replication is, however, complicated by the confounding effects of its helper virus. For example, the MRN complex which plays important roles in ATM-mediated DNA damage responses is degraded in the context of Ad infection [8], and has been shown to be inhibitory to AAV transduction and replication [67]. It is possible that loss of MRN during AAV/Ad co-infection limits the ATM response during AAV infection. The primary role played by ATM following MVM infection is similar to that reported following infections with other viruses such as human papillomavirus HPV [68], SV40 [10], [11] and HSV [12], [69]. It will be interesting to determine if MVM-induced signaling is altered by murine adenovirus co-infection.

DNA damage response signaling is initiated by sensor proteins which recruit transducer kinases and mediators to damage sites [36], [38]. In MVM-infected cells we observed redistribution of MRN complex proteins from their diffuse nuclear localization seen in mock-infected cells into APAR bodies, the sites of ongoing viral replication. Similar accumulation of MRN constituents at sites of viral replication has also been observed during replication of SV40 and AAV [11], [67]. Redistribution of DNA-PKcs, Ku86 and Ku70 has also been observed during AAV replication [31], [32]. These DNA damage proteins may be re-localized as a result of interactions with parvovirus replication proteins [70], [71] or through binding to elements in the virus genome [67]. Although we observed accumulation of Mre11 at the distinct APAR bodies early in MVM infection, we also observed that Mre11 levels were significantly diminished at late time points. The loss of Mre11 was likely due to proteasome-mediated degradation, since it was partially reversed with the proteasome inhibitor MG132. Mre11 has been shown to be inhibitory to the replication of the parvovirus AAV [67], and one of the helper functions provided by Ad is the targeting of the MRN complex for degradation [8], [67]. Proteasome-mediated Mre11 loss has also been observed at late time points during infection with the small DNA tumor virus, SV40 [11]. Surprisingly, the levels of Nbs1 protein, another member of the MRN complex, remained unchanged during MVM infection. The discriminatory loss of a single component of the MRN complex has been previously reported to occur following HSV infection [72], however, in that case Mre11 loss was not proteasome-mediated. Since MRN is required for robust ATM signaling [73], it is possible that it plays important roles early in MVM infection to activate signaling but must be degraded at late times due to an inhibitory function.

Our data suggests that the full spectrum of DNA damage responses during MVM infection required ongoing viral DNA replication. It will be of interest to determine which specific replication intermediates provoke the DDR. Supplying non-replicating input MVM genomes at low MOIs was not sufficient to activate damage signaling. Ectopic expression of NS1 by itself was found to result in a slight but reproducible inductioin of signaling events, including phosphorylation of H2AX. NS1 expression is known to be cytotoxic to cells [74]. The NS1 protein has nickase activities and has been suggested to cause nicks in cellular chromatin [23]. In a recent report the NS1 protein of the closely related parvovirus H1 was shown to cause apoptosis via induction of reactive oxygen species [28]. Any of these NS1 functions could mediate induction of a DDR upon high level expression. Whether these effects are involved in the DDR seen during viral infection is not yet known.

Inhibition of the ATM kinase led to reduced accumulation of MVM replication products and intermediates, suggesting that signaling events enhance viral replication and are exploited by the virus to its benefit. Similar observations have been made with the papovaviruses SV40 [10], [11], JCV [75], polyomavirus [9], and HPV 16 [68], as well as HSV [12]. It is not known if enhancement of MVM replication by ATM signaling events is direct or indirect. For the polyomaviruses, ATM kinase activity contributes directly to increased viral replication, at least in part *via* phosphorylation of large T antigen on S120 [10]. Since we observed accumulation of ATM in MVM APAR bodies, it is possible that ATM and NS1 interact at these sites. ATM phosphorylates several proteins on well characterized S/TQ sites [76], and four of these motifs are present in NS1. The analogous AAV Rep78 protein seems to be phosphorylated by DNA-PK [32]. It will be interesting to determine whether ATM phosphorylates NS1 and whether this contributes to MVM replication.

MVM infection has been reported to bring about cell cycle arrest at G2/M [24]. Here we extend those findings by showing that ATM catalytic activity is required to allow MVM-induced cell cycle arrest. Inhibition of virus replication by the ATM inhibitor could in turn lead to an abrogation of the virally-induced cell cycle block. Alternatively, or in addition to a potential direct effect of ATM on the main MVM replication protein, ATM might contribute to MVM infection by providing an environment suitable for prolonged viral replication [77]. MVM replication does not commence until cells enter S-phase, yet within infected cells MVM replication continues longer than the duration of normal S-phase (Tullis and Pintel, data not shown). Induction of the DDR results in a halt in cellular DNA replication, yet MVM replication persists. It may be that the ATM kinase activity contributes to MVM replication, at least in part, by governing an extended G2 arrest that facilitates prolonged MVM replication. Thus, MVM induction of the DDR would simultaneously inhibit cellular replication and provide an environment suitable for its own replication in host cells. Similar conclusions have recently been suggested for JCV infection [75]. It is perhaps not surprising that smaller DNA viruses, which encode fewer accessory proteins that might counteract a deleterious DDR, have evolved to exploit the DNA damage response for their replication.

## Materials and Methods

### Cell lines

Murine A9, EL4, S49 and human 324K cells were propagated as previously described [78]. NIH3T6 cells were kindly supplied by Kathy Spindler (University of Michigan) and were maintained in DMEM with 5% heat-inactivated bovine serum. Chinese hamster ovary (CHO), AA8 and DNA-PK null V3 cell lines were kindly supplied by Dr David Chen (University of Texas) and were maintained in DMEM with 10% bovine serum.

### Cell synchronization and drug treatments

A9 cells were para-synchronized in G0 by isoleucine deprivation (described in [79]). Hydroxyurea (Sigma) was used at a final concentration of 2 mM, caffeine (Sigma) was used at a final concentration of 2.5 mM, InSolution ATM kinase inhibitor (KU 55933) was obtained from Calbiochem and used at a final concentration of 7.5 µM, DNA-PK inhibitor (NU 7026) was obtained from Sigma and used at a final concentration of 10 µM, MG132 was obtained from Calbiochem and used at a final concentration of 10 µM, and controls were treated with vehicle (DMSO). Cells were pretreated with the inhibitors for 1 hr before infection and for the duration of infection unless otherwise indicated.

### Plasmids and transfections

The NS1 plasmid, NS2 plasmid, and the NS1/2 plasmid which generates a single, slower migrating isoform of NS2 have all been previously described [80]. Transfection of A9 cells was performed using Lipofectamine (Invitrogen) as previously described [78].

### Viruses and infections

Wild-type MVMp, MVMi, and MVM1989 were propagated in 324K cells and titred by plaque assay as previously described [81]. Infections were carried out at an MOI of 10 unless otherwise indicated. Where indicated, re-infection was blocked by addition of neutralizing antibody to the media. UV-inactivation of wild-type MVMp was carried out by exposure of virus to 900 mJ of UV radiation using a GS gene linker UV chamber (BioRad).

### Antibodies

Commercially available antibodies used in this study were obtained from Cell signaling (Mre11, cat # 4895; Nbs1, cat # 3002; p53-P-S15, cat # 9284; Chk1-P-S345, cat # 2348; Chk2-P-T68, cat # 2662; ATM, cat # 2873), Millipore (γH2AX, cat # 05-636; Chk2, cat # 05-649), Santa Cruz (p53, cat # sc-6243), Thermo Fisher (DNA-PKcs, cat # MS-423-P1), Rockland (ATM-P-S1981, cat # 200-301-400), GeneTex (RPA32, cat # GTX 70258), Bethyl (RPA32-P-S4/8, cat # A300-245A), Sigma (Tubulin, cat # T4026) and Abcam (Actin, cat # ab8226). Additional antibodies used for immunofluorescence were obtained from Novus (Nbs1, cat # NB100-143; Nbs1-P-S343, cat # NB100-284A3), Epitomics (ATM, cat # 1549-1; ATM-P-S1981, cat # 2152-1), R&D systems (γH2AX, cat # AF2288), Neomarkers (DNA-PKcs, cat # MS-370-P1), Santa Cruz (Ku70, cat # SC-9033; Ku86 cat # SC-5280) and Genetex (Mre11, cat # GTX70212). The mouse monoclonal antibody against RPA32 was a gift from Tom Melendy (SUNY-Buffalo). All secondary antibodies were from Invitrogen. Other antibodies used include: a polyclonal rabbit antibody raised to the NH2-terminus of NS1/2, a polyclonal rabbit antibody to NS1 (91W12), a polyclonal rabbit antibody to NS2 and a mouse monoclonal antibody to NS1 (CE10) kindly provided by Carol Astell (University of British Columbia).

### Immunoblot analysis

Cells grown and infected in 60 mm dishes were harvested and lysed in modified RIPA buffer containing 20 mM Tris HCL pH 7.5, 150 mM NaCL, 10% glycerol, 1% NP-40, 1% sodium deoxycholate, 0.1% SDS, 1 mM EDTA, 10 mM trisodium pyrophosphate, 20 mM sodium fluoride, 2 mM sodium orthovanadate and 1× protease inhibitor cocktail (Sigma). Alternatively, cells were lysed in 2% SDS lysis buffer as previously described [78]. Protein concentrations were quantified by Bradford assay and equal amounts of lysates were loaded in wells and used for western blot analyses as previously described [78].

### Immunofluorescence

For immunofluorescence, NB324K cells or para-synchronized A9 cells were grown on glass coverslips in 24-well plates and infected with MVMp using an MOI of 10. After 16–24 hr, cells were washed with PBS, fixed with 4% paraformaldehyde for 15 min and extracted with 0.5% Triton X-100 in PBS for 10 min. Nuclei were visualized by staining with DAPI (4′,6′-diaminido-2-phenylindole). The coverslips were mounted in Fluoromount-G (Southern Biotech) and images were acquired using a Leica TCS SP2 confocal microscope. All images were captured using an objective of 63×.

### Analysis of viral DNA

Cell pellets from 60 mm dishes were split in two, with one half used for western blot analysis and the other half for Southern blot analysis. Southern blots were carried out as previously described [56], using whole MVM genome probes.

### Cell cycle analysis

Cells were harvested and fixed in 4% formaldehyde for 15 min at room temperature. Alternatively, cells were fixed in 70% ethanol for 15 min on ice. Cells were then pelleted, washed in PBS and resuspended in 50 µg/ml propidium iodide solution containing 0.1 mg/ml RNAase A as well as 0.05% Trition X-100 for 40 min at 37°C. Cells were resuspended in PBS and flow cytometry was performed using FACScan (BD biosciences). Data were analyzed using Flowjo software (Tree Star, OR).
